# Pathophysiological associations between maternal immune activation and neurodevelopmental disorders in offspring: a comprehensive review

**DOI:** 10.3389/fendo.2025.1681190

**Published:** 2025-10-22

**Authors:** Qing Tang, Xiaofang Wang, Fanqian Yang, Lvyuan Liang, Yuxin Li, Wenbo Liu, Rongyi Zhou, Bingxiang Ma

**Affiliations:** ^1^ Pediatrics Hospital, The First Affiliated Hospital of Henan University of Chinese Medicine, Zhengzhou, Henan, China; ^2^ School of Pediatrics, Henan University of Chinese Medicine, Zhengzhou, Henan, China; ^3^ Hebei University of Chinese Medicine, Shijiazhuang, China

**Keywords:** maternal immune activation, neurodevelopment, endocrine, neurodevelopmental disorders, mechanism, immune response

## Abstract

**Background:**

Maternal immune activation (MIA), triggered by infectious or non-infectious inflammatory stimuli, is a critical risk factor for offspring neurodevelopmental disorders (NDDs) and has become a major focus in neurodevelopmental pathology research.

**Objective:**

This study systematically examined the cellular and molecular mechanisms by which MIA disrupts fetal neurodevelopment, aiming to clarify its impact on NDDs susceptibility and to provide a basis for basic research and clinical intervention.

**Methods:**

A literature search was conducted in PubMed, Web of Science, and Scopus up to June 30, 2025. Both animal and human studies were included, while irrelevant or non-mechanistic reports were excluded. Reference lists of key articles were also screened manually to supplement the database search.

**Results:**

MIA induces systemic elevation of inflammatory cytokines that cross the placenta, activate fetal immune responses, and impair brain development. It suppresses neural stem/progenitor cell proliferation and accelerates premature differentiation, disrupts neuronal migration, alters deep-layer neuron density, and impairs GABAergic interneuron migration. These changes cause neurogenesis and cortical layering abnormalities, increasing the risk of NDDs in offspring, such as autism spectrum disorder (ASD), attention deficit hyperactivity disorder (ADHD), and schizophrenia (SCZ).

**Conclusion:**

Inflammatory cytokines mediate MIA-induced disruptions in neural stem cell proliferation, differentiation, and migration, constituting the main mechanism of maternal impact on fetal neurodevelopment. This insight provides a basis for early diagnosis and precise prenatal intervention to reduce NDDs incidence and improve prognosis.

## 1 Introduction

Maternal immune activation (MIA) denotes a systemic inflammatory response in pregnant individuals induced by infectious or non-infectious immune challenges. Since the 1957 influenza pandemic in Finland revealed an association between mid-gestational infection and schizophrenia in offspring (1), numerous evidence has confirmed that both infectious (2, 3) and non-infectious (4–6) factors during pregnancy can induce MIA and affect fetal neurodevelopment. In recent years, multiple studies have demonstrated a strong correlation between elevated maternal inflammatory cytokine levels during mid-gestation and abnormal social behavior and executive functioning in offspring (7–9). The impact of MIA is mainly mediated by the secretion of inflammatory cytokines (e.g., IL-6, IL-1β, TNF-α), which activate the fetal central immune system and disrupt typical brain development and cortical architecture (10, 11). These cytokines can traverse the placental barrier, affect the fetal brain, activate microglia, and result in neuroinflammation, aberrant neuronal development and migration, and cortical lamination defects (12), thus elevating the risk of neurodevelopmental disorders (NDDs) such as autism spectrum disorder (ASD), attention-deficit/hyperactivity disorder (ADHD), and schizophrenia (SCZ) (13, 14).Elucidating the precise mechanisms by which MIA affects fetal neurodevelopment may provide novel insights into the multifactorial etiology of NDDs (15) and guide early detection and intervention strategies for at-risk populations (16).

A literature search was conducted in PubMed, Web of Science, and Scopus up to June 30, 2025, using combinations of the terms maternal immune activation, fetal neurodevelopment, neural stem cells, neuronal migration, blood–brain barrier, and microglia. Both animal and human studies were included, while irrelevant or non-mechanistic reports were excluded, and the reference lists of key articles were also screened manually to supplement the database search. This study systematically reviews recent basic and clinical research on the effects of MIA on offspring neurodevelopment, aiming to comprehensively summarize its mechanisms and related pathological changes, deeply elucidate the critical roles of MIA in the pathogenesis of neurodevelopmental disorders, and provide theoretical foundations and research directions for early screening, diagnosis, and intervention of related diseases, thereby promoting scientific progress and clinical translation in this field.

## 2 Immunological mechanisms associated with MIA

### 2.1 MIA-induced fetal immune activation

**Figure 1:**
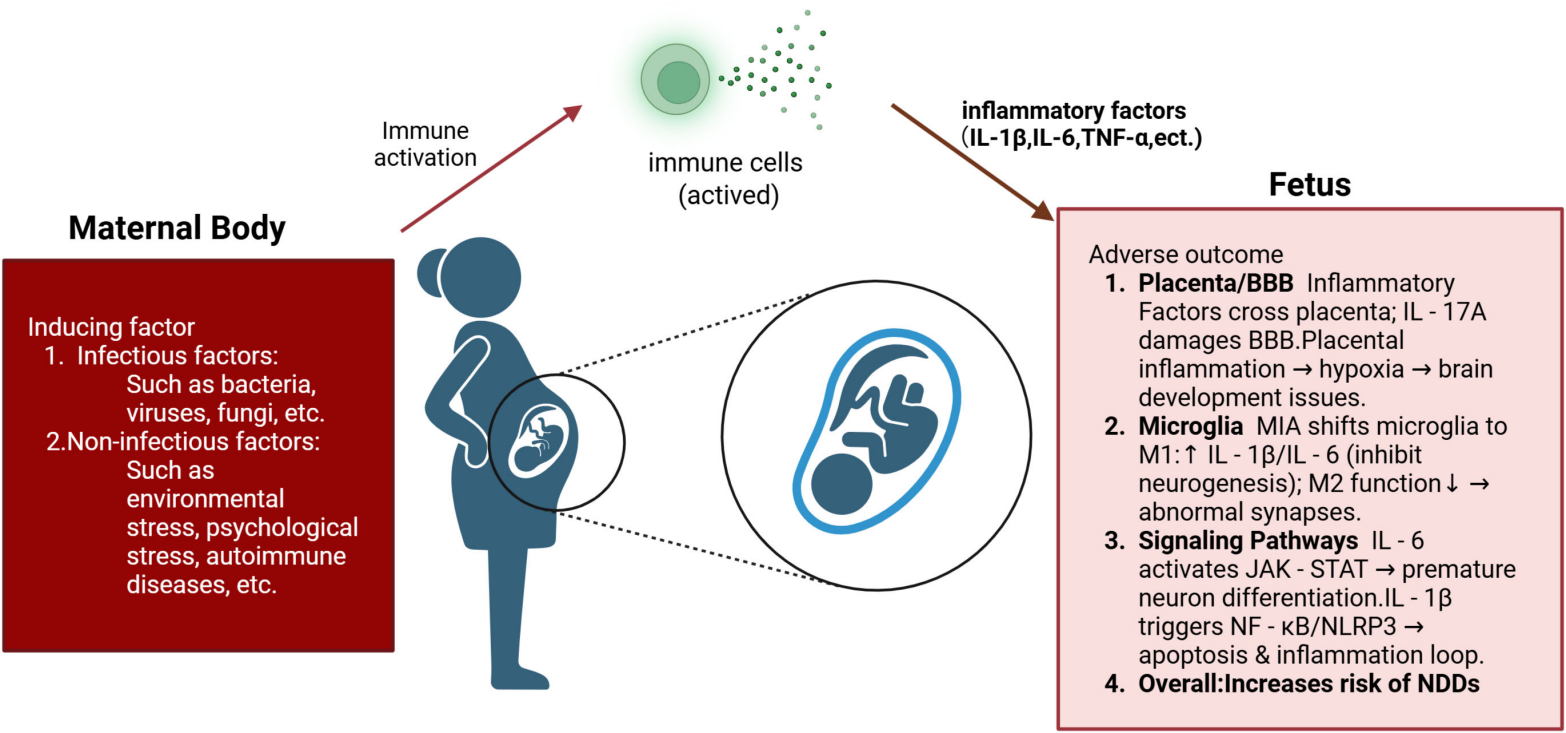
Mechanisms underlying abnormal fetal CNS development induced by MIA. MIA can be triggered by infectious agents (e.g., bacteria, viruses, fungi) or non-infectious stimuli (e.g., environmental stress, psychological stress, autoimmune diseases), leading to systemic maternal immune system activation and the release of abundant inflammatory cytokines capable of crossing the placental barrier. Placental inflammation induces local hypoxia, thereby impairing nutrient and oxygen delivery to the fetus, while IL-17A further compromises the integrity of the fetal BBB. Within the fetal environment, these cytokines drive microglial polarization toward a pro-inflammatory M1 phenotype—characterized by upregulated secretion of IL-1β and IL-6—while suppressing the homeostatic M2 phenotype, consequently impairing synaptic pruning and neurogenesis. Concurrently, IL-6–mediated activation of the JAK–STAT signaling pathway accelerates premature neuronal differentiation, and IL-1β–induced activation of the NF-κB/NLRP3 inflammasome cascade promotes apoptotic processes and chronic neuroinflammation. The convergence of these signaling pathways disrupts the tightly regulated sequence of fetal CNS development, thereby increasing NDDs susceptibility. (CNS, Central nervous system; MIA, Maternal immune activation; BBB, Blood–brain barrier; NDD, Neurodevelopmental disorder; IL, Interleukin; JAK, Janus kinase; STAT, Signal transducer and activator of transcription; NF-κB, Nuclear factor kappa-light-chain-enhancer of activated B cells; NLRP3, NOD-, LRR- and pyrin domain-containing protein 3).

MIA occurs when infectious factors (e.g., bacteria, viruses, fungi) or non-infectious stimuli (e.g., environmental stress, psychological pressure, autoimmune diseases) induce an overactivation of the maternal immune system, leading to the release of abundant inflammatory factors, including pro-inflammatory cytokines and chemokines. For example, pregnant rodents or non-human primates are administered immune stimulants such as lipopolysaccharide (LPS) or polyinosinic:polycytidylic acid [Poly(I:C)] to mimic bacterial or viral infections that activate the maternal immune system (17). Following the onset of MIA, these inflammatory mediators can directly cross the placenta into the fetal circulation, and can also indirectly stimulate further production of inflammatory factors by activating immune cells within the placenta, such as decidual cells (18). Inflammatory cytokines including IL-6, IL-1β, and IL-17A can penetrate the fetal blood-brain barrier (BBB) and enter the central nervous system (CNS), thereby affecting brain neurodevelopment (19) (**Figure 1**). Furthermore, placental inflammation may restrict nutrient and oxygen supply, causing fetal hypoxia and oxidative stress, which further disrupt brain development (20).

### 2.2 MIA-induced dysregulation of fetal central immune cells

After MIA occurs, pro-inflammatory cytokines and chemokines infiltrating the fetal entral nervous system (CNS)may activate microglia, disrupt the balance of M1/M2 differentiation, affecting proliferation of neural progenitor cells and synaptic pruning, leading to abnormal CNS development. Microglia, as specialized immune cells of the CNS, physiologically facilitate neuronal differentiation, modulate synaptic connections, and maintain CNS homeostasis by clearing cell debris and apoptotic cells (21).Microglia exhibit functional heterogeneity, which is commonly described in terms of M1 and M2 polarization states. The M1 phenotype, representing the classically activated pro-inflammatory state, is typically induced during the early stages of neuroinflammation or injury and is characterized by the secretion of pro-inflammatory mediators that exacerbate inflammation and neuronal damage (22). In contrast, the M2 phenotype corresponds to the alternatively activated anti-inflammatory state, which predominates during repair phases or homeostatic conditions. M2 microglia secrete neurotrophic factors such as insulin-like growth factor-1 (IGF-1), support remyelination, facilitate the clearance of inflammatory mediators, and contribute to the maintenance of developmental homeostasis during embryogenesis (23).MIA induces polarization of fetal microglia toward the pro-inflammatory M1 phenotype, disturbing the dynamic balance with anti-inflammatory M2 microglia. This enhances pro-inflammatory responses, impairs reparative functions, and triggers uncontrolled neuroinflammation that disrupts neural development (24).

In the Poly(I:C)-induced MIA model, offspring microglia upregulate CD86 and iNOS expression and secrete abundant pro-inflammatory cytokines IL-1β and IL-6 (25). These pro-inflammatory cytokines suppress neural progenitor cell proliferation, thereby impeding neural development. Moreover, MIA downregulates anti-inflammatory cytokines IL-10 and TGF-β, compromising M2 microglia function (11). M2 microglia are crucial for synaptic pruning and their dysfunction results in aberrant pruning, decreased PV+ interneurons in the adult prefrontal cortex, and disrupted brain function (26).

Besides microglia, meningeal macrophages are also present in the CNS. These cells mainly participate in BBB formation and maintenance (27) and may contribute significantly to MIA-mediated immune responses in the fetal CNS. Nonetheless, research on their specific functions is limited, and the underlying mechanisms are yet to be elucidated.

### 2.3 MIA-mediated dysregulation of fetal inflammatory signaling

Inflammatory cytokines generated by MIA, such as IL-6, IL-1β, and IL-17A, can penetrate the fetal CNS and activate signaling pathways including JAK-STAT and NF-κB. These activations disrupt neural stem cell (NSC) differentiation and impair BBB integrity, ultimately leading to abnormal fetal CNS development. Notably, IL-6 activates the JAK-STAT signaling cascade in NSCs (28, 29). IL-6 interacts with the membrane-bound IL-6 receptor (mIL-6R), triggering the activation of JAK kinases and phosphorylation of STAT proteins (30, 31). This signaling cascade downregulates PAX6 expression and promotes premature differentiation of radial glial cells (RGCs) into neurons, thereby disrupting NSCs self-renewal and the normal course of neurogenesis (32, 33). IL-1β engages IL-1R1 receptors in the fetal brain, activating the NF-κB signaling pathway (34), which stimulates microglia to produce reactive oxygen species (ROS) and TNF-α, leading to damage of neural progenitor cells, apoptosis, and interference with normal neural development. Moreover, IL-1β can activate the NLRP3 inflammasome, which activates caspase-1 to cleave pro-IL-1β into active IL-1β, creating a positive feedback loop that exacerbates inflammation and adversely affects neuronal development (35, 36). IL-17A uniquely contributes to MIA by promoting the phosphorylation of tight junction proteins such as Claudin-5 in fetal brain endothelial cells via the placenta (37). This compromises BBB integrity, facilitates peripheral immune cell infiltration, and exacerbates neuroinflammation, ultimately exerting harmful effects on the fetal nervous system.

## 3 Impact of MIA on fetal neural development

### 3.1 Physiological cortical neurodevelopment

**Figure 2:**
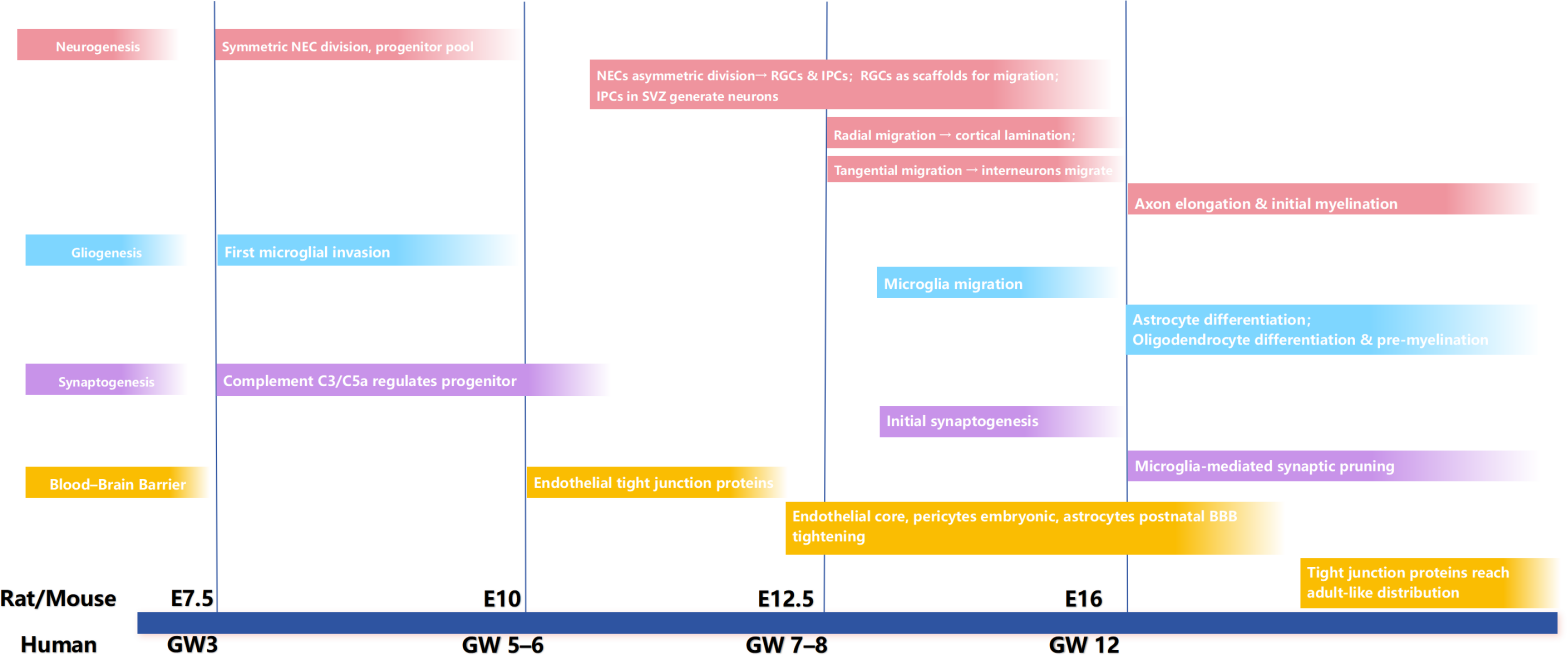
Timeline of CNS development in rodents and humans. CNS development follows a tightly regulated timeline, shown here with rodent (E) and human (GW) milestones. Neurogenesis starts with symmetric NEC division (rodent E7.5, human GW3) to build a progenitor pool. Then, NECs undergo asymmetric division (rodent E10–12.5, human GW5–8), generating RGCs and IPCs in the subventricular zone. Radial migration enables cortical lamination, while tangential migration guides interneuron movement. Later (rodent E16+, human GW12+), axons elongate and initial myelination occurs. Gliogenesis involves early microglial invasion (rodent E7.5, human GW3), subsequent microglia migration, and later astrocyte/oligodendrocyte differentiation plus pre-myelination. Synaptogenesis is regulated by complement C3/C5a for progenitors initially, followed by initial synaptogenesis and microglia-mediated synaptic pruning. BBB development begins with endothelial tight junction proteins, forms an endothelial core with embryonic pericytes and postnatal astrocyte-mediated tightening, and finally achieves adult-like tight junction protein distribution. (CNS, Central nervous system; E, Embryonic days; GW, Gestational weeks; NEC, Neuroepithelial cell; RGC, Radial glial cell; IPC, Intermediate progenitor cell; BBB, Blood–brain barrier).

In typical cortical development, multiple stages of neural cell proliferation can be observed, as exemplified in rodent embryogenesis (**Figure 2**). During the early embryonic period (E7.5–E10), neuroepithelial cells (NECs) expand through symmetric division to generate a sufficient number of cells. At this stage, microglia first infiltrate the developing brain, where they regulate the local microenvironment, while complement factors such as C3 and C5a exhibit spatiotemporal expression patterns that contribute to the proliferation of neural progenitor cells (26, 38). From approximately embryonic day 11 to 16 (E11–E16), NEC division shifts toward asymmetric division, giving rise to RGCs. These RGCs undergo further asymmetric divisions to produce one RGC and one intermediate progenitor cell (IPC), a process referred to as direct and indirect neurogenesis, respectively, which underlies the generation of all cortical neurons and several glial lineages (39). RGCs serve as both neuronal progenitors and scaffolds for the migration of newborn neurons; whereas IPCs, primarily located in the subventricular zone (SVZ), can generate neurons through limited symmetric divisions (40, 41). Overall, proper proliferation and differentiation of neural cells are closely related to the number and volume of neurons in the developing cortex.

Neuronal migration is a fundamental process in normal brain development, with its precision being crucial for the establishment of cortical functional areas and neural networks. During radial migration, newly generated neurons migrate along radial glial fibers in an “inside-out” manner (e.g., layer VI forms first, followed by layers II-III), ensuring the orderly lamination of the cortical plate from TBR1+ (deep layers) to CUX1+ (superficial layers). This process depends on adhesion molecules such as N-cadherin and guidance cues from the Reelin signaling pathway, forming the foundation for cortical column organization (42, 43). In contrast, tangential migration is responsible for guiding GABAergic interneurons from the ganglionic eminence (GE) into the cortex.

A uniform distribution of these inhibitory neurons is essential for maintaining cortical excitation–inhibition balance. Additionally, other newborn neurons also undergo tangential migration within the cortex to form functional columns or specific nuclei, such as the amygdala and striatum (44).

After E12.5, neural progenitor cells predominantly undergo asymmetric division, and newly generated neurons migrate from the ventricular zone to form the cortical plate. Interneurons migrate from the ventral to the dorsal regions in response to chemokine signaling. Microglia, which begin to widely colonize the lateral cortex around E14, support neurogenesis during this stage but transiently disappear from the cortical plate at E15–E16 to avoid interfering with neuronal maturation (45, 46). Following the completion of neuronal migration, from late embryonic stages to birth (E17–P0), astrocytes and oligodendrocytes begin to differentiate, contributing to blood–brain barrier (BBB) formation and subsequent myelination. During this period, neurons establish initial synaptic connections, while excessive synapses are pruned through complement-mediated processes and microglial phagocytosis to optimize neural circuits. In parallel, axons extend and gradually undergo myelination, thereby enhancing the efficiency of signal transmission and consolidating cortical functional architecture (26, 38, 44).

As neural cell proliferation, differentiation, and neuronal migration are completed, the CNS enters the stage of functional maturation from late embryogenesis through the postnatal period, during which the BBB undergoes a coordinated, multi-stage developmental process involving multiple cell types. In humans, telencephalic vascularization begins around gestational week 8. By week 12, cerebral endothelial cells express tight junction proteins such as occludin and claudin-5, which restrict the entry of endogenous albumin into the brain. By week 18, the distribution of these proteins approximates that observed in adults (47–49). Endothelial cells form the structural core of the BBB, while pericytes promote tight junction formation during embryogenesis, and astrocytes contribute to barrier “tightening” after birth in rodents (50–52). Functional maturation of the BBB is further enhanced by the increased expression of efflux transporters, although regional heterogeneity and interspecies differences exist. Moreover, maternal factors may influence BBB development, thereby linking maternal environment to fetal neurodevelopment (53).

### 3.2 Impact of MIA on fetal neurodevelopment

#### 3.2.1 MIA reduces proliferation of fetal neural cells

**Figure 3:**
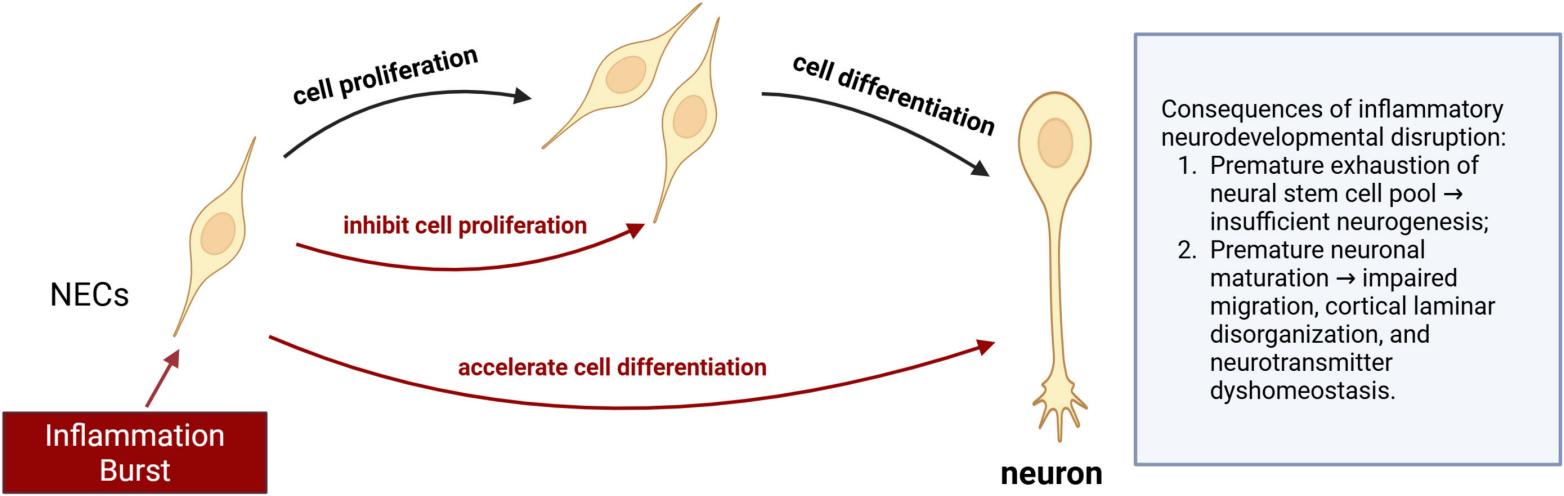
Impact of MIA on fetal neurodevelopment. An acute perinatal “inflammation burst” triggered by MIA exerts two interrelated effects on NECs, 1. Elevated pro-inflammatory cytokines inhibit NEC proliferation, reducing the pool of progenitor cells required for subsequent neurogenesis. 2. The same cytokine milieu accelerates differentiation of remaining NECs into immature neurons, leading to premature depletion of the NSC reservoir. These alterations result in insufficient neuronal output, aberrant cortical lamination, and neurotransmitter imbalance. Long-term consequences include deficits in cortical laminar organization, impaired interneuron integration, and disrupted E/I balance, all of which contribute to ASD, ADHD, and other NDDs. (MIA, Maternal immune activation, NEC, Neural epithelial cell; NSC, Neural stem cell; E/I balance, Excitatory–inhibitory balance; ASD, Autism spectrum disorder; ADHD, Attention deficit hyperactivity disorder; NDD, Neurodevelopmental disorder).

At specific stages of fetal neurodevelopment, MIA significantly suppresses the proliferation of NECs, characterized by a shortened cell cycle. The reduced proliferation of NECs leads to premature depletion of the NSC pool, resulting in insufficient generation of neurons and impairing subsequent cortical layering and expansion (**Figure 3**). In MIA offspring animal models, cortical thinning or abnormal thickening is observed, along with disrupted laminar organization and cellular density, which affects cognitive functions in the offspring. Animal studies have shown that following MIA induction at embryonic days 12–13, the number of phospho-histone H3 and Ki67-positive cells in the fetal mouse cortex is significantly reduced (54, 55), indicating impaired proliferative capacity of NECs. This proliferative defect is likely closely related to the effects of inflammatory cytokines. For example, IL-6 can activate the STAT3 signaling pathway, thereby downregulating the expression of cyclin D1 and reducing the frequency of cell division (56). Thymidine analog labeling experiments in LPS-induced MIA models showed a 22% reduction in S-phase cells within 24 hours. This directly demonstrates restricted DNA synthesis in offspring and further confirms the inhibitory effect of MIA on NEC proliferation (28).

#### 3.2.2 MIA accelerates differentiation of fetal neural stem cells

MIA promotes premature exit of NSCs from the proliferative state and accelerates their differentiation into neurons, leading to early neuronal maturation and impaired normal migration (**Figure 3**). This results in cortical layering disruption and disturbances in neurotransmitter homeostasis, such as abnormal dopamine levels in the offspring’s prefrontal cortex. The division pattern of RGCs changes, with a significant increase in neurogenic divisions (56). Meanwhile, the expansion of TBR2-positive IPCs in the SVZ is inhibited, affecting the indirect neurogenesis pathway (57). This shift in differentiation is closely linked to inflammation-induced epigenetic remodeling. Studies have found that upregulation of histone acetyltransferase CBP activity promotes the expression of neurodifferentiation genes, thereby driving NSC differentiation toward neurons (33).

#### 3.2.3 Disrupted neuronal migration alters the density of deep cortical neurons

**Figure 4:**
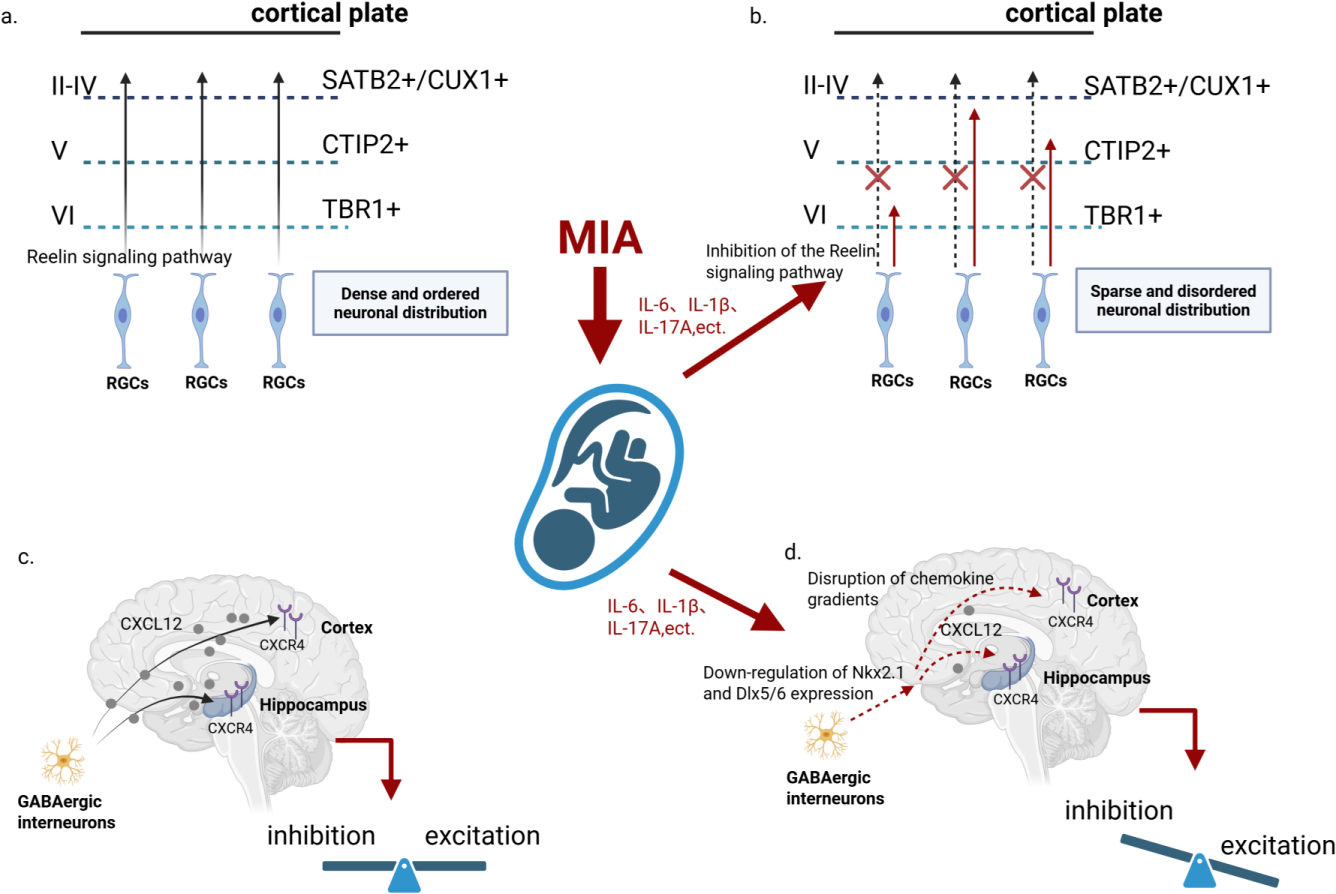
Fetal neuronal migration abnormalities triggered by MIA. **(a)** Normal cortical development (Control): Radial glial cells (RGCs) secrete Reelin to establish accurate guidance cues, enabling the orderly migration of cortical neurons—TBR1^+^ neurons into layer VI, CTIP2^+^ into layer V, and SATB2^+^/CUX1^+^ into layers II–IV—resulting in a dense and well-structured cortical plate. **(b)** Reelin signaling disruption following MIA exposure: Pro-inflammatory cytokines (IL-6, IL-1β, IL-17A) inhibit the Reelin signaling pathway, impairing RGC-derived guidance cues. Neuronal migration fails to reach proper laminar destinations (red ✗), leading to cortical disorganization and reduced neuronal density. **(c)** Normal chemokine-guided interneuron migration (Control): GABAergic interneurons migrate from the ventral forebrain to the cortex and hippocampus along CXCL12–CXCR4 chemokine gradients, contributing to the establishment of E/I balance. **(d)** MIA-induced disruption of chemotactic and transcriptional programs: Inflammatory signals downregulate transcription factors Nkx2.1 and Dlx5/6 and impair CXCL12–CXCR4 gradient formation, resulting in abnormal interneuron migration. This disturbance disrupts E/I balance and predisposes cortical circuits to functional deficits. (MIA, maternal immune activation; RGC, radial glial cell; IL, interleukin; E/I balance, excitatory–inhibitory balance; CXCL12, C-X-C motif chemokine ligand 12; CXCR4, C-X-C motif chemokine receptor 4; Nkx2.1, NK2 homeobox 1; Dlx5/6, Distal-less homeobox 5/6; TBR1, T-box brain gene 1; CTIP2, COUP-TF-interacting protein 2; SATB2, special AT-rich sequence-binding protein 2; CUX1, cut-like homeobox 1.).

MIA causes profound disruption to the typical lamination pattern of the cortical plate, triggering premature differentiation of deep-layer neurons and migration abnormalities in upper-layer neurons. These alterations ultimately lead to neuronal mispositioning, disordered cortical expansion, and laminar disorganization (58) (**Figure 4**). Experimental evidence indicates that in the offspring of MIA model rats, the density of TBR1+ (layer VI) and CTIP2+ (layer V) neurons is altered, and SATB2+ (upper-layer) neurons exhibit disorganized distribution (59, 60). These abnormalities are tightly linked to alterations in the temporal window of neurogenesis. In addition, overexpression of maternal IL-6 suppresses the fetal Reelin signaling pathway, a key mechanism guiding neuronal migration via radial glial fibers, and its downregulation exacerbates lamination abnormalities in the cortex (29).

#### 3.2.4 Impaired migration of GABAergic interneurons

MIA specifically affects the migration of GABAergic interneurons derived from the medial ganglionic eminence (61, 62). This disruption impairs the excitation-inhibition balance in brain regions such as the cortex and hippocampus (**Figure 4**). The resulting imbalance contributes to network hyperactivity or dysregulation, a hallmark of neurodevelopmental disorders including ASD and schizophrenia (63, 64). Evidence from Poly(I:C)-induced MIA models reveals reduced expression of Nkx2.1 and Dlx5/6, which disturbs the migratory trajectories of interneuron precursors and leads to their aggregation in the cortical intermediate zone (34). The observed migratory defect is linked to inflammation-driven disruption of chemokine gradients. For instance, CXCL12 guides the directed migration of GABAergic interneurons through CXCR4, and MIA interferes with this gradient, causing aberrant interneuron migration (65).

### 3.3 Stage-specific effects of MIA on neurodevelopment

The impact of MIA on offspring neurodevelopment is highly time-dependent and closely linked to the vulnerability of specific developmental stages of the fetal brain. In murine MIA models, early gestation (E9) coincides with the peak of neurogenesis. At this stage, MIA disrupts neural progenitor cell proliferation and differentiation, leading to a general reduction in embryonic brain volume by E18, with particularly pronounced effects in the hippocampus, globus pallidus, and cerebellum. In parallel, apoptotic cell density is markedly increased, indicating that early insults are dominated by suppressed proliferation and enhanced programmed cell death (8, 66). During mid-gestation (E12.5–E13.5), when large-scale neuronal migration toward target cortical regions is underway, MIA interferes with neuronal migration and cortical lamination. This results in migration delays, abnormal migratory trajectories, accumulation of deep-layer cortical neurons, and a relative reduction of superficial-layer neurons. Moreover, overall neuronal proliferation is reduced, differentiation is prematurely induced, and the layered cortical structure is disrupted, ultimately predisposing to later network abnormalities (67). In late gestation (E17), the brain enters a stage of synaptic pruning and circuit refinement. MIA at this stage triggers robust neuroinflammation, resulting in abnormal enlargement of the embryonic brain at E18, particularly in the hippocampus. This is accompanied by a marked increase in the density of pathological neurons and glial cells characterized by ultrastructural condensation induced by stress, inflammation, or oxidative damage, suggesting that oxidative stress and inflammatory injury dominate late-stage pathology, while dysregulation of maternal anti-inflammatory mechanisms may exacerbate these effects (68, 69).

### 3.4 Interactions between MIA and the developing Blood–Brain Barrier

The interaction between the BBB and MIA is fundamentally rooted in the developmental vulnerability of the BBB. Structurally, the embryonic BBB is immature, with relatively low expression of tight junction proteins in vascular endothelial cells (e.g., claudin-5, ZO-1) and incomplete pericyte coverage, resulting in inherently higher permeability (49). In rodents, BBB formation begins around embryonic day 13 (E13), with pericytes playing a pivotal role in the maturation of tight junctions. If MIA occurs during this critical developmental window (e.g., E12.5–E15), it may precisely target these vulnerabilities, allowing maternal inflammatory cytokines such as IL-6, IL-17, and TNF-α to more readily penetrate the immature BBB and enter the fetal CNS, thereby laying the foundation for subsequent injury (70).

Moreover, MIA disrupts BBB development through a cascade of interrelated processes with long-lasting consequences. Initially, maternal cytokine storms induced by MIA permit inflammatory mediators to enter the fetal circulation and activate microglia, which subsequently release COX2-derived prostaglandin E2 (PGE2). PGE2 impairs pericyte function and upregulates VCAM1 expression, while MIA simultaneously reduces tight junction protein levels, collectively increasing BBB permeability (70, 71). Elevated BBB permeability is detectable immediately after MIA exposure and can persist into adulthood, facilitating continuous entry of inflammatory mediators into the CNS. This chronic neuroinflammatory state, driven by sustained glial activation, ultimately disrupts normal neurodevelopment (71, 72).

## 4 Underlying mechanisms of MIA involvement in NDDs

### 4.1 Pathogenic mechanisms of ASD mediated by MIA

ASD is a neurodevelopmental disorder with early developmental onset, characterized clinically by persistent deficits in social interaction and communication, as well as restricted, repetitive patterns of behavior, interests, or activities. Epidemiological studies have shown that maternal infections during pregnancy (e.g., influenza, toxoplasmosis) and elevated inflammatory markers (e.g., influenza, toxoplasmosis) are significantly associated with increased risk of ASD (73). Maternal infections during pregnancy induce MIA, which activates maternal Th17 cells and pro-inflammatory cytokines (e.g., IL-6, IL-17A). This activation disrupts placental barrier function, resulting in fetal immune activation and abnormal brain development (14). Studies have demonstrated that MIA reduces fetal Reelin protein expression and causes neuronal migration abnormalities, which further result in cortical lamination defects (74). These abnormalities may be related to atypical brain structural development in ASD. Furthermore, MIA induces fetal immune activation and immune cell dysregulation, resulting in aberrant synaptic pruning and synaptic dysfunction (75). This process is mediated by proinflammatory cytokines that cross the placenta and activate fetal microglia and astrocytes, creating a chronic neuroinflammatory milieu. Dysregulation of microglial CX3CR1–CX3CL1 and complement signaling leads to region- and sex-specific defects in synaptic elimination, while aberrant MHCI upregulation further disrupts synapse formation and selection. Collectively, these immune-driven alterations impair excitatory–inhibitory balance and weaken synaptic transmission, providing a mechanistic link between MIA and long-term neurodevelopmental abnormalities (76, 77). Both of these effects are associated with social behavior deficits and repetitive behaviors (78), potentially representing key mechanisms by which MIA contributes to ASD pathogenesis.

### 4.2 Pathogenic mechanisms of ADHD mediated by MIA

ADHD is a prevalent chronic neurodevelopmental condition marked by inattentiveness, hyperactivity, and impulsivity. It commonly begins in childhood and substantially impairs learning, daily activities, and social interactions. Perinatal infections, maternal obesity, and maternal psychological stress are all significant risk factors for ADHD (79). The occurrence of ADHD induced by MIA may involve aberrant migration of ventral midbrain dopaminergic neurons and decreased dopaminergic projections in the prefrontal cortex, manifesting as inattention and impulsivity (80). Synaptic plasticity deficits result in hippocampal long-term depression (LTD) abnormalities, which impact learning and executive functions (81). Additionally, MIA-induced oxidative stress and metabolic dysfunction worsen dopaminergic neuronal injury, contributing to the core symptoms of ADHD (82).

### 4.3 Pathogenic mechanisms of schizophrenia mediated by MIA

Schizophrenia is a severe psychiatric disorder of unknown etiology, commonly accompanied by abnormalities in perception, thought, emotion, and behavior, often with a protracted course. Infection during late pregnancy (e.g., influenza) raises schizophrenia risk by about three times and shows synergistic effects with genetic vulnerability (83). MIA results in aberrant neuronal migration and cortical disarray, interfering with neurotransmitter system stability. This disruption potentially impairs synaptic plasticity and working memory, thereby contributing to positive symptoms (hallucinations) and negative symptoms (emotional blunting) (84). Additionally, hyperdopaminergic activity in the mesolimbic pathway correlates with hallucinations and cognitive disorganization (85). Moreover, impaired neurodevelopment disrupts neural stem cell proliferation and differentiation, reduces dendritic spine density, and compromises synaptic plasticity, thereby diminishing hippocampal neurogenesis and contributing to septum pellucidum enlargement, which in turn underlie negative symptoms such as emotional blunting (86, 87).

## 5 Conclusion

MIA is a pathological state of maternal inflammation during pregnancy that can disrupt fetal neurodevelopment through multiple mechanisms. These mechanisms include activation of the fetal immune system, modulation of central immune cells, and interference with inflammatory signaling pathways. MIA releases inflammatory cytokines such as IL-6, IL-1β, and IL-17A, which cross the placental barrier to activate the fetal CNS immune system, disrupt microglial M1/M2 polarization and related inflammatory pathways. This results in inhibited proliferation of fetal NSCs, accelerated differentiation, and abnormal neuronal migration. Ultimately, these changes cause dysregulated neurogenesis, cortical lamination abnormalities, and impaired neural circuit development, manifesting as NDD phenotypes including ASD, ADHD, and SCZ. Overall, MIA has become a significant environmental risk factor for NDDs. Future studies should clarify the specific roles of individual inflammatory mediators during critical developmental windows and evaluate targeted interventions against inflammatory signaling to enable early identification and precision prevention for high-risk pregnancies. Ultimately, this will provide a solid theoretical foundation and effective strategies to reduce NDD incidence and improve prognosis.
